# Targeting the sigma-1 receptor with pridopidine induces functional neurorestoration in spinal cord ischemia-reperfusion injury

**DOI:** 10.1007/s00210-025-03851-3

**Published:** 2025-02-12

**Authors:** Eman Sweed, Suzan A. Khodir, Shaimaa Mohamed Motawea, Hala El-Haron, Basma Abdelnaby Mostafa, Mona S. Elkholy, Mohammud Salim, Doaa Z. M. Shebl

**Affiliations:** 1 Clinical Pharmacology Department, Faculty of Medicine, Menoufia University, Menoufia, Egypt; 2 Clinical Pharmacology Department, Menoufia National University, Menoufia, Egypt; 3 Medical Physiology Department, Faculty of Medicine, Menoufia University, Menoufia, Egypt; 4 Medical Physiology Department, Menoufia National University, Menoufia, Egypt; 5 Histology and Cell Biology, Faculty of Medicine, Menoufia University, Menoufia, 32511 Egypt; 6 AlRyada University for Science and Technology, Menoufia, 32511 Egypt; 7 Medical Biochemistry and Molecular Biology Department, Faculty of Medicine, Menoufia University, Menoufia, Egypt; 8 Neuropsychiatry Department, Faculty of Medicine, Menoufia University, Menoufia, Egypt; 9 Neurosurgery Department, Faculty of Medicine, Menoufia University, Menoufia, Egypt; 10 Medical Biochemistry, Menoufia National University, Menoufia, Egypt

**Keywords:** Pridopidine, Sig-1R, BDNF, GDNF, Caspase-3, Spinal cord IRI

## Abstract

Spinal cord ischemia reperfusion injury (IRI) occurs with an incidence of 1–32%, often leading to paraplegia with limited prevention options. Pridopidine (Prdpn), a highly selective sigma-1 receptor (Sig-1R) agonist, serves as a protein chaperone that is engaged in neuroplasticity and cellular defense. This research aimed to assess the neuroprotective properties of Prdpn in spinal cord IRI in rats and investigate the underlying mechanisms. Forty male Wistar albino rats were randomly allocated into 4 groups: control, sham, IRI, and IRI + Prdpn. Tarlov’s test was used to examine behavioral performance, as well as withdrawal from agonizing stimuli and the placing/stepping reflex (SPR). Biochemical markers, including spinal malondialdehyde (MDA), AOPP, antioxidant GPX, TNF-α and IL-1β, and apoptotic caspase-3, were measured, along with BDNF, GDNF, and Sig-1R gene expression. Histopathological changes in spinal cord tissue were also evaluated. Spinal cord IRI significantly caused neurological deficits, evidenced by lower scores in Tarlov’s test, withdrawal from agonizing stimuli, and SPR. Biochemically, spinal cord IRI led to decreased GPX and increased MDA, AOPP, TNF-α, IL-1β, caspase-3, and GDNF levels, along with downregulated BDNF and Sig-1R gene expression. Histopathologically, spinal cord IRI resulted in greater spinal neuronal degeneration, apoptosis, and demyelination. However, treatment with Prdpn significantly improved behavioral outcomes and partially reversed the biochemical and histopathological alterations. Prdpn improved spinal cord IRI-induced behavioral deficits through its antioxidant, anti-inflammatory, anti-apoptotic, and neurotrophic properties. It suggests promise as a potential treatment option to stop spinal cord IRI.

## Highlights

- Pridopidine, a Sig-1R agonist, produces functionally relevant neuroprotective and neurorestorative influences in this spinal cord IRI rat model.
- The antioxidant, Anti-Apoptotic, anti-inflammatory and neurotrophic properties of pridopidine mediate its neuroprotective effects.

## Introduction

Spinal cord ischemia-reperfusion injury (IRI) is a catastrophic side effect of endovascular aortic repair and thoracoabdominal aneurysm surgery. It can also arise from spinal tumors, degeneration, or trauma. It causes severe motor and sensory impairment (Xie et al. 2024). About 5–18% of patients still experience spinal cord IRI, despite technological advancements and surgical competence (Xie et al. 2024). Paraplegia places a severe psychological and financial strain pressure not just on the sufferers and their families but also on society (Anjum et al. 2020). Currently, the available methods of stopping the progression of paraplegia are inadequate (Tian et al. 2023). Thus, to improve the outcomes of patients with spinal cord IRI, it is imperative to scrutinize its cellular and molecular causes and develop useful treatment options.

Spinal cord IRI primarily comprises two phases: ischemia and reperfusion. Ischemia occurs when major arteries become blocked or narrowed. Reperfusion causes cell death by producing reactive oxygen species (ROS) and inflammatory mediators (Sánchez 2019). Excitotoxicity, nitric oxide-provoked degeneration of neurons, elevated calcium within cells, eicosanoid synthesis, inflammatory processes, apoptosis, and damage from free radicals all contribute to neurologic harm following IRI. These mechanisms ought to be considered as parallel channels that operate consecutively (de Haan et al. 2001).

In ischemia-reperfusion settings, an excess of ROS and oxygen free radicals can cause severe lipid peroxidation, as well as the destruction of proteins and DNA with reduced capacity for both enzymatic and nonenzymatic antioxidant defense (George et al. 2024). Moreover, ischemia-induced oxidative stress alters a variety of genes and signaling pathways. Nitric oxide synthase (iNOS), the primary enzyme that induces the conversion of L-arginine into nitric oxide (NO), has been observed to exhibit elevated levels of neurotoxicity or neuroprotection in brain tissues that have undergone IRI (Roy et al. 2023). Furthermore, a high level of proinflammatory cytokine production may cause neuronal death or apoptosis (Wang et al. 2017). So, an efficient decrease of oxidative damage, apoptosis, and inflammatory processes could be critical for neuroprotection in spinal cord IRI.

Following spinal cord ischemia, axonal regeneration has been achieved through increasing the amount of neurotrophic factors (Elmalky et al. 2024). These factors have been shown to enhance neuronal preservation, axonal development, and central nervous system (CNS) repair (Liu et al. 2022). Among them, the effects of glial derived neurotrophic factor (GDNF) and brain-derived neurotrophic factor (BDNF) on the promotion of motor axon outgrowth after spinal cord damage have been thoroughly investigated (Hu et al. 2023). Progenitor cells can proliferate and differentiate into mature oligodendrocytes with the help of BDNF and GDNF. Moreover, they support the myelin basic protein (MPB) expression and axonal regeneration in SCI (Zhao et al. 2022).

A ligand-activated chaperone protein known as the sigma-1 receptor (Sig-1R) is widely distributed in mitochondrial-associated endoplasmic reticulum membranes and supports various processes of cell protection and survival (Munguia-Galaviz et al. 2023). Interestingly, the nervous system’s tissue expression profile of Sig-1R indicates that it is mostly expressed in spinal motor neurons and the brainstem (Ionescu et al. 2019). Sig-1R agonists have been demonstrated to have effects on neural restoration and protection in several models of acquired brain injury and neurodegenerative conditions (Penke et al. 2018).

Numerous pathways have been suggested to describe these advantageous effects, such as transcription factor and gene expression regulation, upregulation of genes that prevent apoptosis, reduction of microglial activation, reduction in ROS and NO production, and elevation of trophic factor secretion. Moreover, it controls cell survival and ER-mitochondria calcium (Ca^2+^) signaling (Li et al. 2024). In fact, loss of Sig-1R in mice causes motor abnormalities (Couly et al. 2022).

Pridopidine (Prdpn), a new chemical, is being investigated to treat Parkinson’s disease (PD), Huntington’s disorder (HD), and amyotrophic lateral sclerosis (Brimson et al. 2020). There is growing evidence that Prdpn’s therapeutic actions are mediated through its high-affinity binding to Sig-1R at pharmacologically relevant doses (Munguia-Galaviz et al. 2023). By stimulating the Sig-1R receptor, Prdpn improves damaged spine density and abnormal intracellular Ca^2+^ signaling in HD neurons (Ryskamp et al. 2017).

Prdpn therapy stimulates neural restoration in the nigrostriatal system and dramatically enhances the dopaminergic fiber density and nigral dopamine cell bodies in the 6-hydroxydopamine (6-OHDA) lesion mice models of PD (Francardo et al. 2019). Prdpn’s neuroprotective effects are mediated through Sig-1R, as they were eliminated in mice lacking Sig-1R (Francardo et al. 2019). It enhances BDNF production in neurons via the Sig-1R pathway and increases striatal BDNF and GDNF expression in PD mouse models, effects that are also Sig-1R dependent (Francardo et al. 2019, Ryskamp et al. 2019).

To our knowledge, the potential role of Prdpn in spinal cord IRI has not been examined. Consequently, this study aimed to explore for the first time the protective effects of Prdpn in spinal cord IRI mediated via Sig-1R activation through reducing apoptosis, oxidative stress, and inflammation together with upregulation of neurotropic factors.

## Material and methods

The current study was authorized by the ethics committee of the Faculty of Medicine, Menoufia University, Egypt, and was conducted in perfect conformity with the rules governing the use and care of lab animals. Our study used a randomized controlled animal experimental strategy, Faculty of Medicine, Menoufia University, Egypt and consented to by the national ethics committee with IRB No 9-2024 BIO9. The experiment followed the standards for Animal Research: Reporting of In Vivo Experiments (ARRIVE) (Percie du Sert et al. 2020). G*Power was used for estimating sample size with power 80% and confidence interval 95%.

### Animals and experimental groups

This study used 40 mature male Wister albino rats (aged 2–3 months, each one weighing 160–200 g). Four rats were kept in each of the completely ventilated, 80 × 40 × 30 cm cages, with regular light/dark cycles, room temperature, and unrestricted accessibility to water and a semi-synthetic balanced diet. They were left for 1 week for acclimatization before starting the experiment.

Four equal experimental groups of ten rats each were randomly assigned:

1. Control group

Normal rats received a single subcutaneous (s.c.) injection of 1 ml normal saline once every day for 15 days, along with unlimited accessibility to water and regular meal.

2. Sham group

Rats were given a single s.c. injection of 1 ml of normal saline once a day for 15 days with unrestricted access to water and a consistent diet. On day 14, the same surgical techniques, which are detailed below, were applied to the animals in order to produce spinal cord IRI. However, the aorta was not clamped.

3. Spinal cord IRI group

Before the surgical induction of spinal cord ischemia-reperfusion injury (IRI), rats received 1 ml of normal saline once a day via s.c. injection for 14 days, in addition to having unrestricted access to water and a regular meal, as explained below. Following a 24-h surgical procedure, rats again received 1 ml of normal saline via s.c. injection.

4. Pridopidine-treated spinal cord ischemia-reperfusion injury group (IRI + Prdpn)

#### Rats received Prdpn

Before the spinal cord IRI being induced, Prdpn (Prilenia, Gooimeer, Naarden, Netherlands) was dissolved in physiological saline right prior to being used and administered at a volume of 0.1 ml/10 g body weight in one single s.c. injection each day at a dose of (0.3 mg/kg) (Francardo et al. 2014; 2019) for 14 days, as explained below.

### Induction of spinal cord ischemia-reperfusion injury

After the start of the experiment by 14 days, rats were exposed to IRI consistent with an approach adopted by Lafci et al. (2013). Initially, rats received intramuscular injections of 50 mg/kg BW of ketamine (Amoun, Al-Qalyubia, Egypt) and 5 mg/kg BW of xylazine (Biomeda, Dublin, Irelanda), without endotracheal intubation or mechanical ventilation, then, If necessary, a half-dose of ketamine during the surgery. The rectum was provided with rectal probe to monitor the rat’s body temperature, which was regulated by a thermal pad that operates at 37 to 38 °C. Every animal underwent sterile processing.

Following rat stabilization, the retroperitoneum was exposed, and a standard middle incision was used to perform a laparotomy and reach the abdominal aorta.

Ischemia of spinal cord was provoked by compressing the aorta for 30 min with small aneurysm vascular clamp (El-sahaba Company, Egypt) between just below the origin of the two renal arteries and just above to the aortic division. The clamp was then removed, and a 5/0 polypropylene suture was used to close the anterior abdominal wall.

Fusidic acid, a local antibiotic from Minapharm in Egypt, was put into the lesion and cefotaxime (Eipico, Egypt) at a dose of 10 mg/kg body weight was administered intraperitoneally. In a plastic box, the animals were left to recuperate for 3 h at a temperature of 28 °C. They were then put in different enclosures with unrestricted accessibility to water and nutrition.

### Neurological assessment

When the experimental period ended (16 days after the experiment began and 48 h following IRI initiation in the concerned surgical groups), neurological behavior was assessed. The rat’s hindlimb motor dysfunction was evaluated by assigning a number between 0 and 5 to each rat in accordance with Tarlov’s scoring criteria (Tarlov’s Scoring System, Tarlov, 1972). The criteria were as follows: Scores of zero indicate lacking voluntary movement of the hindlimbs; 1 indicates noticeable joint movement; 2 indicate moving actively but has incapacity to sit with no assistance; 3 indicate sitting ability but has incapacity to hop; four indicate feeble hop; and five indicate full hindlimb function restoration (Morsy et al. 2010).

Similar to this, the sensory function of one hindlimb’s withdrawal from a painful stimulus was evaluated using a scoring system of 0–1, where 0 denotes no response to a noxious stimulus and 1 denotes a withdrawal response. Each rat’s placing/stepping reflex (SPR) was evaluated via dragging its hind paw’s dorsum along a surface procedure’s edge. In this test, this typically induces a synchronizing lifting and placement reply that was classified using the following scores: zero for normal, one for weak, and two for no stepping (Morsy et al. 2010; Bashir et al. 2020).

### Sample preparation and biochemical measurements

Subsequently, rats were killed by intravenously administering a large dose of sodium thiopental for humanitarian purposes, and spinal cord samples were promptly extracted out of the third, fourth, and fifth lumbar segments. After being gathered, these tissue samples underwent homogenization. Spinal cord tissues were weighed between 300 and 500 mg prior to homogenization, and they were properly washed in frozen PBS (0.02mol/L, pH 7.0–7.2) to eliminate any extra blood.

The tissues were chopped into tiny particles and mixed in 500 μl of PBS with an ice-filled glass homogenizer. The homogenates were then centrifuged at 5000 rpm for 15 min, and the supernatant was taken out and kept at −20 °C for measurement of malondialdehyde (MDA) and glutathione peroxidase (GPX) that were measured by spectrophotometric using available commercial kits (Biodiagnostic CO., Dokki, Giza, Egypt). Advanced oxidation protein products (AOPP) (MBS930313, MyBioSource, Sandiego, CA, USA), spinal cord TNF-α (MBS2507393, MyBioSource, Sandiego, CA, USA), rat prostaglandin E2 (PGE2) (MBS262150, MyBioSource, Sandiego, CA, USA), rat IL-1β (ab100768, Abcam, Cambridge, UK), rat caspase 3 (CASP3) (MBS018987, MyBioSource, Sandiego, CA, USA), rat GDNF (ab213901, Abcam, Cambridge, UK) were assessed using rat enzyme linked immunosorbent assay (ELISA) consistent with manufacturer commands. A segment of the lumber spinal cord was used to evaluate BDNF and sigma-1 receptor ligand gene expression.

The remaining portion of the removed lumbar spinal cord was washed with PBS and preserved in 10% formalin saline. The tissue was fixed for 48 h and then washed under running water for 4 h. Following gradient dehydration, the tissue was immersed in xylene and subsequently embedded in paraffin. Finally, slices were cut with a paraffin slicer to generate 7-µm-thick paraffin slices.

These sections were used for histopathological examination by light microscope utilizing hematoxylin and eosin staining (H&E) for detection of the number of the anterior horn’s motor neuron count.

### Quantitative assay of sigma-1 receptor and BDNF gene expression via RT-PCR

Spinal cord tissues were processed for entire RNA isolation utilizing the Qiagen RN easy together with universal kit from the USA. RNA quality and purity were then guaranteed. RNA was kept at −80 °C until it was used. After that, the initial step was cDNA production by means of QuantiTect Reverse Transcription Kit, Qiagen from the USA, consuming Applied Biosystems 2720 thermal cycler (Singapore) for a single cycle only. GAPDH primers were manipulated in RT-PCR reactions as a control for RNA loading. The second step was cDNA amplification: cDNA was consumed in SYBR green-based quantitative real-time PCR for relative quantification (RQ) of BDNF gene expression via SensiFASTTMSYBR Lo-ROX Kit, USA, utilizing the next constructed primers (Midland, Texas): The forward primer for sigma-1 receptor was (CTCAGCTTGCTCGACAGTACG), and the reverse primer was (CAGGGTAGACGGAATAACACC). The forward primer for BDNF was (GCTGCCTTGATGTTTACTTTG), and the reverse primer was (ATGGGATTACACTTGGTCTCGT). Finally, data were analyzed using version 2.0.1 of the Applied Biosystems 7500 software. The BDNF gene expression RQ was made via a comparative ∆∆Ct approach, where the target gene mRNA levels are normalized to an endogenous reference gene (GAPDH) and compared to a control.

### Histopathological studies H&E staining

#### H & E staining

Samples from the spinal cord were processed with formal solutions, alcohol, and xylol before being embedded in paraffin blocks. Following that, tissue samples were sliced sagittally using a microtome at a thickness of 5 μm. H&E staining of samples. Under light microscopy, the produced samples were histo-pathologically evaluated.

#### Luxol fast blue (LFB) stain

Rat spinal cord paraffin slices were produced on the 15th day following modeling in every group. The components were then dyed Luxol fast blue. Each set of rats had spinal cord paraffin slices obtained the day following modeling. After attaching to glass slides, slices were dehydrated at 37 °C, washed with 0.01 mol/l PBS, colored overnight in LFB, rinsed with 95% alcohol, and washed again with distilled water following 10 s of 0.05% lithium carbonate differentiation, Sections were rinsed using distilled water. Slices were processed by 70% fine differentiation to separate white and gray matter. After cleaning, dehydrate, clean, and seal portions. Under a light microscope, rat spinal cord tissue was examined for damage and demyelination. Image-Pro Plus (Media Cybernetics, MD, USA) quantified LFB staining (Wang et al. 2024).

### Statistical analysis

Statistical analyses were conducted via IBM SPSS Statistics 27 (IBM, Armonk, NY, USA) and Graph Pad prism 9 (San Diego, CA, USA). One-way ANOVA was consumed for comparing the groups, followed by the post hoc analysis using Tukey test in normally distributed data. Data are revealed as *mean* ± *SD*. The Kruskal-Wallis test was employed for data that was not normally distributed, and the Tukey test was utilized as a post hoc test. A statistically significant *P*-value was defined as less than 0.05.

## Results

### Neurological assessment

**Fig. 1:**
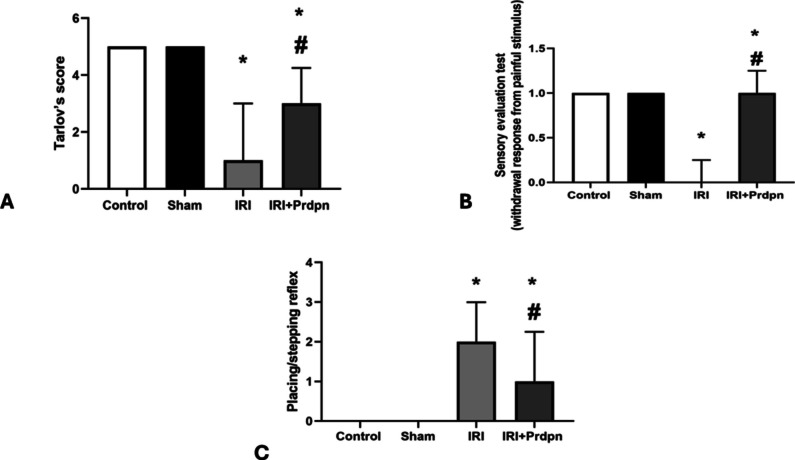
Effect of pridopidine on Neurological assessment: **A** Tarlov’s scoring criteria, **B** withdrawal response from painful stimulus, and **C** placing/stepping reflex (SPR). Data was compared using (Kruskal-Wallis test) and presented in the form of *median* ± *IQR*. *P < 0.05 was significant when IRI group was compared to control and sham groups. ^#^*P* < 0.05 vs. IRI + Prdpn group

The median Tarlov’s score (motor function) significantly decreased in both the IRI (1.00 ± 2.00) and the IRI + Prdpn groups (3.00 ± 1.25) relative to the control and sham groups (both 5.00 ± 0.00). However, the IRI + Prdpn group displayed a significant elevation in Tarlov’s score compared to the IRI group (Fig. 1A).

The sensory evaluation test in the IRI group (0 ± 0.25) indicated a marked deterioration in sensory function relative to the control and sham groups (both 1.00 ± 0.00). In contrast, the IRI + Prdpn group demonstrated a substantial improvement in sensory function (1.00 ± 0.25) compared with the IRI group (Fig. 1B).

For the placing/stepping reflex, the control and sham groups showed normal responses (0.00 ± 0.00), while the IRI group exhibited no response (2.00 ± 1.00), which was drastically distinct from both the sham and control groups. In the IRI + Prdpn group, there was a weak response (1.00 ± 1.25), showing an improvement contrasted to the IRI group (Fig. 1C).

### Biochemical measurements

#### Oxidative stress

There was no meaningful variation in the mean levels of MDA, GPX, and AOPP between the control and sham groups (9.33 ± 1.08, 56.75 ± 1.54 nmol/g·tissue, 118 ± 4.85 nmol/ml vs. 9.66 ± 1.96, 57 ± 2.02 nmol/g·tissue, and 120.33 ± 7.00 nmol/ml, respectively). However, the IRI group displayed a considerable elevation in oxidative stress markers (*P* < 0.05) with values of 61 ± 2.82, 33.75 ± 1.17 nmol/g tissue, and 314 ± 9.89 nmol/ml, contrasted to both the sham and control groups.

**Fig. 2:**
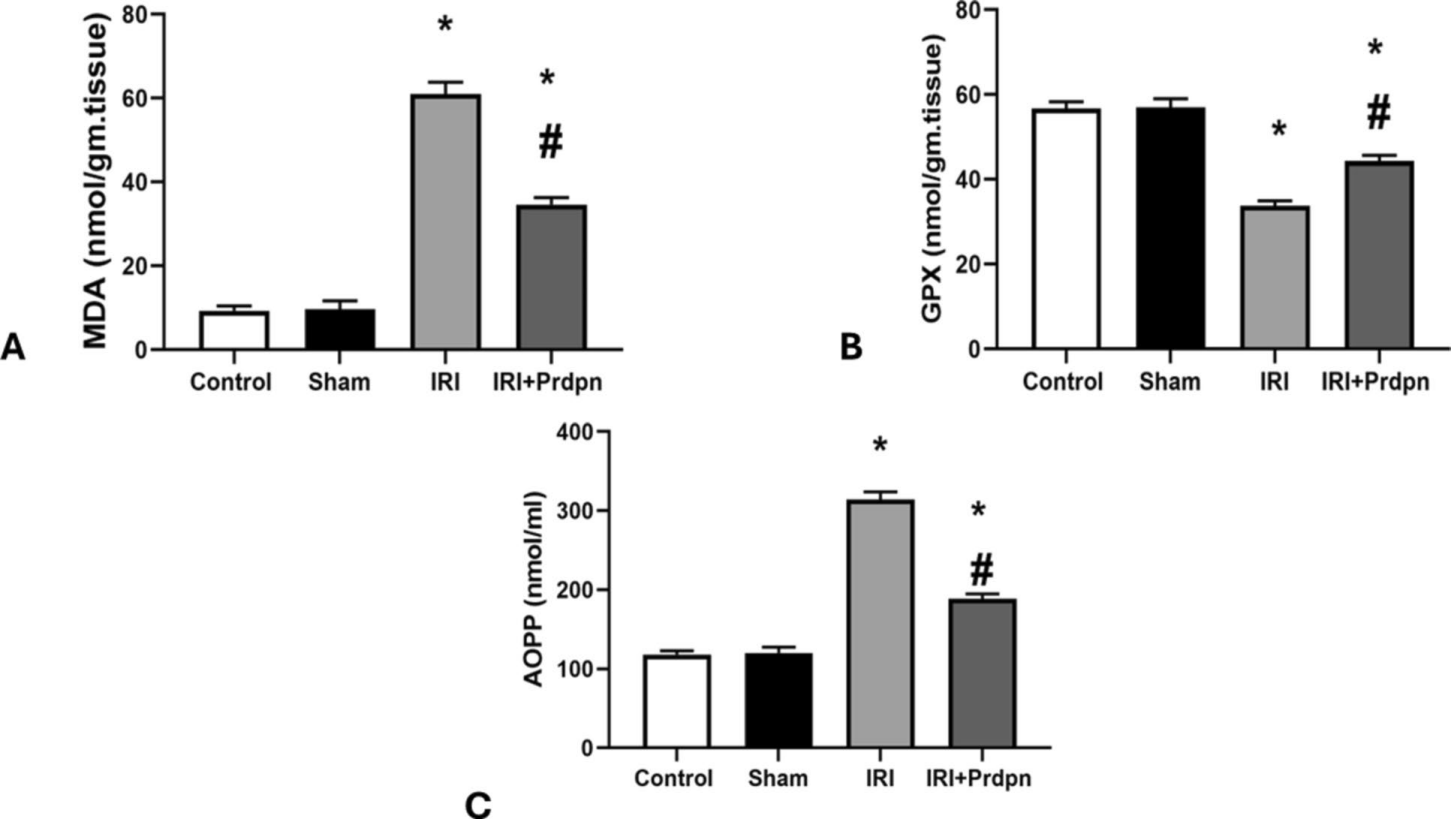
Effect of pridopidine on oxidative stress markers: **A** malondialdehyde (MDA) (nmol/g·tissue), **B** glutathione peroxidase (GPX) (nmol/g·tissue) and **C** advanced oxidation protein products (AOPP) (nmol/ml). Data was compared using (ANOVA test) and presented in the form of *mean* ± *SD*. *P < 0.05 was significant when IRI was group compared to control group. ^#^*P* < 0.05 vs. IRI + Prdpn group

The IRI + Prdpn group revealed a substantial decrease (*P* < 0.05) in MDA, GPX, and AOPP levels (34.66 ± 1.63, 44.33 ± 1.36 nmol/g·tissue, and 188.83 ± 5.91 nmol/ml) compared to the IRI group. However, oxidative stress markers remained significantly elevated (*P* < 0.05) in the IRI + Prdpn group as opposed to both the sham and control groups (Fig. 2A, B, and C).

#### Inflammatory markers

There was no considerable variation in the mean levels of inflammatory mediators (TNF-α and IL-1β) between the control and sham groups (106.66 ± 5.04 ng/l, 84 ± 3.40 pg/ml vs. 108.83 ± 5.41 ng/l, and 84.66 ± 3.93 pg/ml, respectively). However, the IRI group displayed a substantial elevation (*P* < 0.05) in these inflammatory mediators’ levels (321.83 ± 1.94 ng/l and 401.16 ± 5.41 pg/ml) relative to both the sham and control groups.

**Fig. 3:**
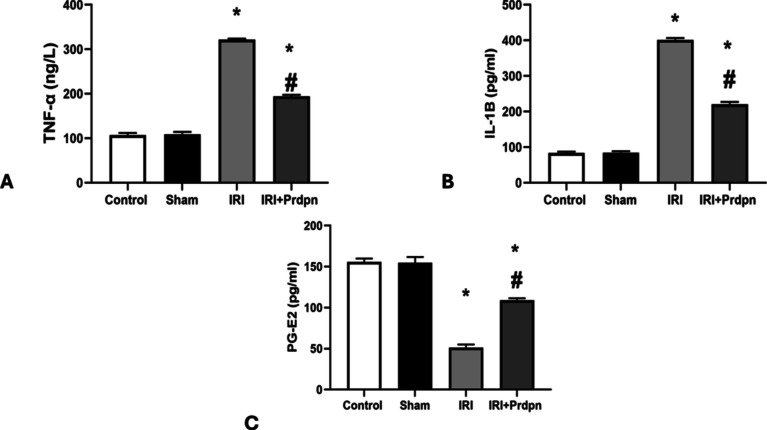
Effect of pridopidine on inflammatory markers: **A** tumor necrosis factor (TNF)-α (ng/l), **B** interleukin (IL)-1β (pg/ml), and **C** prostaglandin (PG-E2) (pg/ml). Data was compared using (ANOVA test) and presented in the form of *mean* ± *SD*. *P < 0.05 significant when IRI group compared to control group. ^#^*P* < 0.05 vs. IRI + Prdpn group

In the IRI + Prdpn group, there was a considerable decline (*P* < 0.05) in the mean levels of inflammatory mediators (194.33 ± 3.50 ng/l and 220.50 ± 6.65 pg/ml) relative to the IRI group. Nevertheless, the levels in the IRI + Prdpn group maintained significantly elevated levels (*P* < 0.05) as opposed to both the sham and control groups (Fig. 3A, B).

There was no significant change in the mean value of PG-E2 between control and sham groups (155.66 ± 4.17 pg/ml vs 154.83 ± 6.91 pg/ml), respectively; however, there was a significant increase (*P* < 0.05) in the mean value of PG-E2 in IRI group (51.33 ± 3.72 pg/ml) compared to control and sham groups. There was a significant decrease (*P* < 0.05) in the mean value of PG-E2 in IRI + Prdpn group (109 ± 2.36 pg/ml) compared to IRI group, while the mean value of PG-E2 showed a significant increase (*P* < 0.05) in IRI + Prdpn group compared to control and sham groups (Fig. 3C).

#### Caspase-3

There was no considerable variation in the mean caspase-3 levels between the control and sham groups (27.50 ± 2.58 vs. 27.66 ± 2.87 ng/ml, respectively). However, the IRI group demonstrated a substantial rise (*P* < 0.05) in caspase-3 levels (80.33 ± 2.42 ng/ml) relative to both the sham and control groups.

**Fig. 4:**
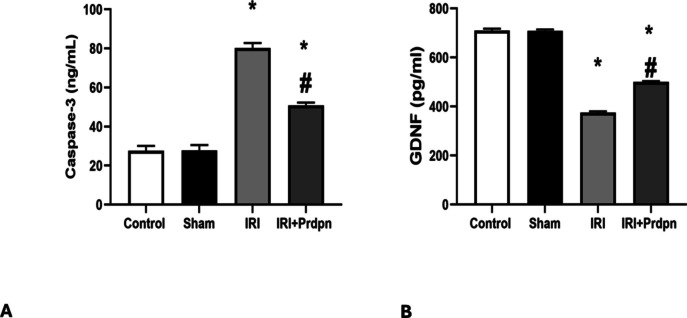
Effect of pridopidine on **A** caspase 3 (CASP3) and **B** glial derived neurotrophic factor (GDNF) (pg/ml): data was compared using (ANOVA test) and presented in the form of *mean* ± *SD*. *P < 0.05 significant when IRI group was compared to control group. ^#^*P* < 0.05 vs. IRI + Prdpn group

In the IRI + Prdpn group, there was a substantial decline (*P* <0.05) in caspase-3 levels (50.83 ± 1.47 ng/ml) compared to the IRI group. Despite this decrease, caspase-3 levels in the IRI + Prdpn group remained drastically higher (*P* < 0.05) relative to the control and sham groups (Fig. 4A).

#### GDNF

There was no considerable variation in the mean GDNF levels between the control and sham groups (710.33 ± 7.11 pg/ml vs. 709 ± 5.40 pg/ml, respectively). However, the IRI group showed a substantial drop (*P* < 0.05) in GDNF levels (375.83 ± 4.44 pg/ml) compared with both the sham and control groups.

The IRI + Prdpn group revealed a substantial rise (*P* < 0.05) in GDNF levels (500 ± 3.46 pg/ml) relative to the IRI group. Despite this increase, GDNF levels in the IRI + Prdpn group remained extensively lower (*P* < 0.05) than both the sham and control groups (Fig. 4B).

#### Sigma-1 receptor and BDNF gene expression

The mRNA expression levels of the Sig-1R and BDNF proteins in the sham group showed no considerable changes relative to the control group.

**Fig. 5:**
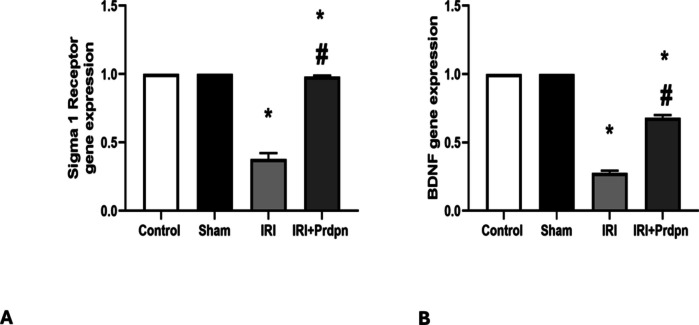
Effect of pridopidine on sigma-1 receptor (**A**) and BDNF gene expression (**B**): data was compared using (ANOVA test) and presented in the form of *mean* ± *SD*. *P < 0.05 significant when IRI group was compared to control group. ^#^*P* < 0.05 vs. IRI + Prdpn group

Conversely, the Sig-1R and BDNF mRNA expression levels in the IRI group (0.37 ± 0.04 and 0.27 ± 0.01, respectively) were substantially lower (*P* < 0.05) than the control group. However, in the IRI + Prdpn group, both the Sig-1R and BDNF expression levels were dramatically upregulated (*P* < 0.05) to 0.98 ± 0.007 and 0.68 ± 0.02, respectively, relative to the IRI group. Despite this increase, the levels remained considerably lower than both the sham and control groups (Fig. 5A, B).

### Histopathological findings

**Fig. 6:**
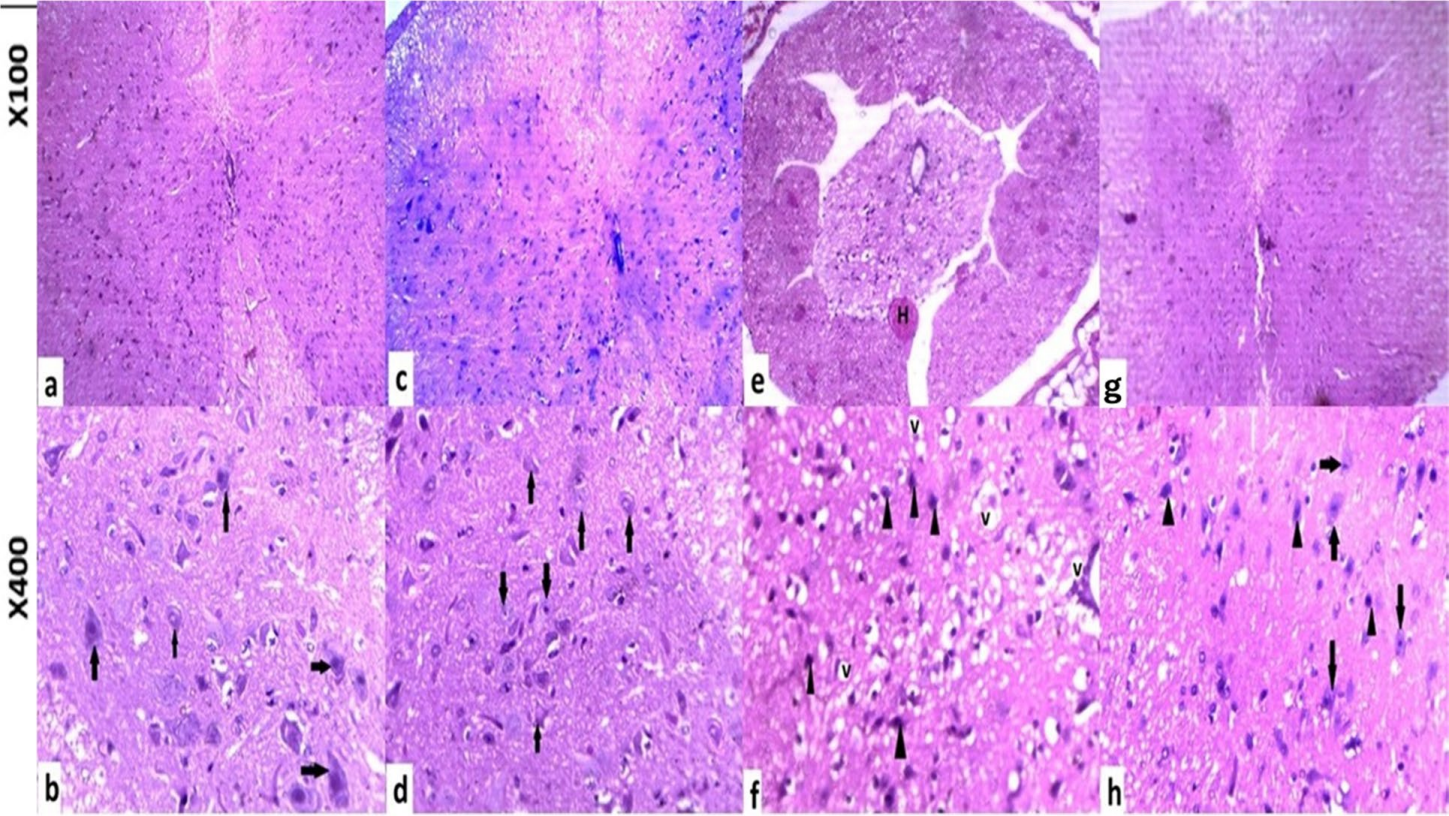
Control (**a**, **b**, **c**, and **d**) and sham groups (**c**, **d**, and **e**) displayed white matter periphery, butterfly-shaped gray matter center, and motor neurons (arrow) with vesicular nuclei and Nissl bodies in the cytoplasm. White and gray matter architecture was disrupted in the ischemic group (**e**, **f**), with dilated spinal canals, massive vacuoles (v), and hemorrhagic foci (H) Nissl substance loss, cell cavitation, and pyknotic darkly stained nucleus characterized damaged tiny neurons (arrowhead). Pridopidone and spinal cord ischemia reperfusion (**g**, **h**) demonstrated H-shaped gray matter, peripheral white matter, recovered spinal canal, and variable vacuolar degradation (C). A few pyknotic neurons (arrow head) were discovered with normal neurons (arrow) having Nissl bodies (H&E)

H&E sections of the spinal cords of the sham and control groups exhibited typical anatomical features, including a white matter periphery and a butterfly-shaped gray matter in the center that housed motor neurons with typical histological characteristics, such as Nissl bodies and vesicular nuclei in the cytoplasm. Additionally, the spinal cords displayed a normal spinal canal. The spinal canal of the ischemic group was dilated, and necrotic tissue manifested as extensive cavities at the site of injury. In addition to hemorrhagic foci, white and gray matter architecture was disrupted. Loss of Nissl substance, cavitation encircling the cells and the presence of pyknotic darkly stained nuclei were indicators of damaged neurons. Variable degrees of vacuolar degeneration were detected in the spinal cord. The degree of injury was noticeably reduced in the group that received Prdpn along with spinal cord ischemia-reperfusion treatment. H-shaped gray matter, peripheral white matter, and the spinal canal have been partially restored. A limited quantity of pyknotic neurons, which served as an indicator of mild to moderate impact, were identified in conjunction with neurons that exhibited a typical histological appearance, including the existence of Nissl bodies in the cytoplasm (Fig. 6).

#### Luxol fast blue stain

**Fig. 7:**
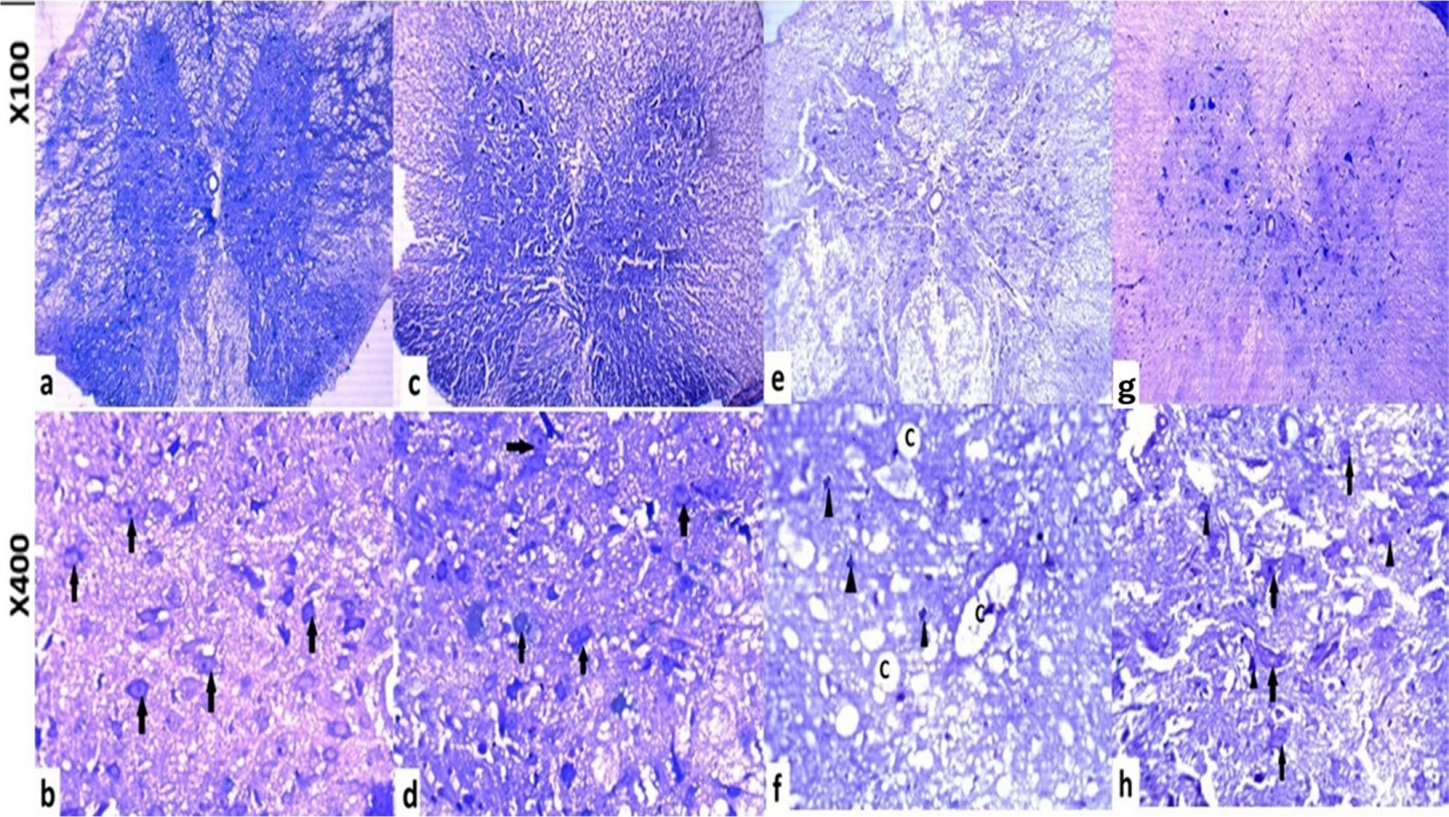
The level of demyelination of spinal cord tissues in the ischemia group was assessed by Luxol fast blue (LFB) staining, which revealed: **a**-**b**) The control group showed normal myelination. **c**-**d**) Sham group. **e**-**f**) IRI group displaying areas of demyelination and cavitation (**c**), small pyknotic neurons (arrowhead), and intact neurons (arrow). **g**-**h**) The IRI+ Prpdn treated group demonstrated enhanced myelination

**Fig. 8:**
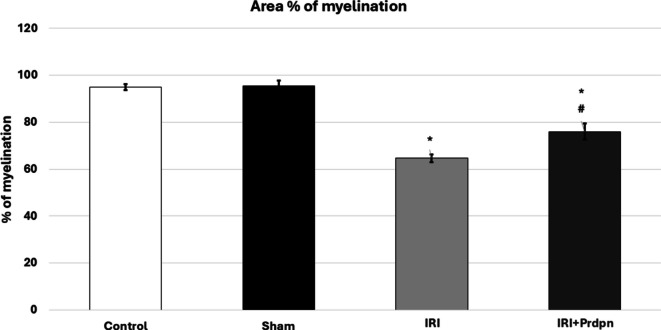
Histogram depicts the mean area percentage of myelination compared to the total area of white matter as assessed by Luxol fast blue staining. Data was compared using (ANOVA test) and presented in the form of *mean* ± *SD*. *P < 0.05 significant when IRI group was compared to control group. ^#^*P* < 0.05 vs. IRI + Prdpn group

There was a significant loss of myelin in the white matter of the ischemic reperfusion groups compared to the sham and control groups (Fig. 8). The dorsal and ventral columns demonstrated cavitation and vacuolation, respectively. Additionally, demyelination and neuronal loss were observed (Figure). In the Prdpn group, myelination and neuronal content increased moderately, while cavitation decreased (Figs. 7 and 8).

## Discussion

After thoracoabdominal aortic surgery, there is a 1–32% chance of spinal cord IRI, which can cause paraplegia and possibly death (Marturano et al. 2022). Primary injury is unavoidable, but secondary injury can be treated. Oxidative stress, lipid peroxidation, apoptosis, and inflammation are among secondary damage processes (Aghili-Mehrizi et al. 2022). Although several treatments were developed for treating the spinal cord following IRI and shielding it from thoracoabdominal aortic injury, there is still a paucity of data supporting the gold-standard spinal cord IRI management (Kuru Bektaşoğlu et al. 2024).

Prdpn is a highly selective and strong Sig-1R agonist (Grachev et al. 2021). The Sig-1R regulates several cellular pathways essential to neuronal function and is found at the interface between the mitochondria and endoplasmic reticulum (ER) (Lenoir et al. 2022). Sig-1R agonists are neuroprotective, antidepressant, and antiamnesic substances (Goguadze et al. 2019). Prdpn activates the Sig-1R, which increases various cell pathways, contributing to neuroprotection, as demonstrated in numerous models of neurodegenerative illness (Wang et al. 2023). Prdpn suppresses neuroinflammation, reduces oxidative stress, improves synaptic connection, inhibits apoptotic pathways, prevents autophagy, and is extremely neuroprotective (Lachance et al. 2023). Moreover, it has been demonstrated to increase the expression of genes that are downstream of the BDNF receptor (Geva et al. 2016). These impacts are clearly operated via the Sig-1R. To our knowledge, this is the first study to examine Prdpn’s effectiveness on spinal cord IRI.

In the rat model, our first observation shows deterioration of motor function, loss of SPR, and sensory impairment. This was demonstrated by lower scores on a neurological assessment of limb taking away from agonizing stimulus, SPR, and Tarlov’s scoring system 48 h after blocking the aorta.

This finding suggested that vascular ischemia caused spinal cord damage, which in turn disrupted many neurological processes (Tang et al. 2009). Additional research by Bashir et al. confirmed our findings. The primary causes of the neurologic dysfunction that follows SCI are the high levels of ROS produced from many sources during the SCI together with the vigorous inflammation caused by invading neutrophils, macrophages, activated microglia, and astrocytes (Bashir et al. 2020). In addition, researchers have shown that spinal cord injury results in disturbance of sensory and motor neurological functions (Zhang et al. 2022).

The findings of our study demonstrated that Prdpn enhanced sensory and motor functions of the rat hind limbs. The behavioral recovery induced by Prdpn dines was obvious by the better scores achieved through the neurological assessment use (SPR, Tarlov’s scoring system, and the limb removal from agonizing stimulus). Animal studies displayed that Prdpn improved defects in axonal transport and reduced neuromuscular junction disturbance, along with motor neuron degeneration in mice suffering from amyotrophic lateral sclerosis. Prdpn activated the prosurvival extracellular signal-regulated kinases (ERK) pathway and generated its advantageous effects via the Sig-1R (Mori et al. 2013). Moreover, the Sig-1R was shown to suppress the generation of ROS and activated microglia (Ionescu et al. 2019). Researchers discovered that administering a Sig-1R agonist substantially enhanced the motor function in spinal cord injury mice’s lower limbs (Tan et al. 2024). Persistent ischemic injury in spinal cord IRI produced a significant level of ROS, resulting in oxidative stress. Excessive oxygen radicals destroyed cell proteins, membrane lipids, and DNA (Kahveci et al. 2021). Prdpn’s motor effects were initially linked to low-affinity antagonism of dopamine D2 receptors (D2Rs). However, in vivo PET studies suggest that behaviorally relevant doses primarily target the sigma-1 receptor (S1R) due to its significantly higher binding affinity for S1R compared to D2R (Francardo et al. 2019).

Our results revealed a dramatic rise in spinal cord homogenate levels of AOPP and MDA, indicators of protein and lipid oxidation, in the rat model of IRI. This correlated with a drop in antioxidant GPX, due to free radical overproduction.

Additional research confirmed our findings. Hazzaa et al. reported that the spinal cord injury led to increased ROS and decreased antioxidants with impaired sensory and motor functions (Hazzaa et al. 2021). Similar outcomes have been documented in spinal cord injuries in rats caused by long-term sciatic nerve constriction injuries (Nishihara et al. 2020). Additionally, there was a correlation between the levels of proinflammatory cytokines, MDA, and AOPP in plasma, indicating that AOPP functioned as a mediator in oxidative stress (Pekárková et al. 2001).

Our results revealed the beneficial impact of Prdpn administration on oxidative stress markers in IRI rats. The Prdpn antioxidant effect was examined by Eddings et al. who stated that Prdpn reversed the effects of Sig-1R knockdown on mitochondrial ROS levels, restored the antioxidant response, and enhanced mitochondrial respiration (Eddings et al. 2019). Furthermore, Geva et al. showed that Prdpn exerted neuroprotective effects through the Sig-1R and was probably linked to controlling ROS levels and mitochondrial activity (Geva et al. 2016). It has been revealed that Sig-1Rs shield cells against oxidative damage. By modulating Nrf2 (nuclear factor erythroid 2-related factor), its induction lowered the formation of ROS, increased endogenous antioxidants, and reduced oxidative stress (Wang et al. 2019).

Our research showed that during IRI there was a substantial rise in the inflammatory markers IL-1β and TNF-α in the spinal cord tissue. Ischemia activated multiple pathophysiologic signaling pathways, including cytokines, which in turn triggered the start of an inflammatory cascade (Abd-Elhafiz et al. 2024).

These proinflammatory cytokines stimulated monocyte migration, macrophage activation, and neutrophil adherence to the injured site (Kahveci et al. 2021). The pathophysiology of ischemia injury in the human brain has been shown to involve COX-2 and its product PGE2 (Deng et al. 2020). Therefore, decreasing these inflammatory mediators is crucial for limiting secondary injury and regenerating the remaining nerve cells as spinal cord IRI progresses.

The findings of our study disclosed that Prdpn could diminish the increasing proinflammatory cytokines TNF-a and IL-1β caused by IRI, implying its anti-inflammatory impacts, which concurred with Francardo et al. (2019). The anti-inflammatory effects of Prdpn were attributed to its ability to inhibit the proinflammatory cytokine generation while improving the anti-inflammatory cytokine IL-10 (Szabo et al. 2014). Sig-1R could prevent T-cell triggering (Wu et al. 2021). Furthermore, a Sig-1R agonist downregulated iNOS and TNF-α. The activation of Nrf2 and heme oxygenase-1 (HO-1) in astrocytes contributed to the anti-inflammatory and antioxidative stress properties of Sig-1R stimulation (Wang et al. 2019).

After spinal cord IRI, apoptotic pathways begin to activate, which causes neuronal loss (Dong et al. 2022). Ischemia occurs when circulation stops, and acute ATP exhaustion promotes cell necrosis (Kuru Bektaşoğlu et al. 2024).

Previous research has established caspase-3 as a valid biomarker for spinal cord IRI, reflecting the extent of apoptotic processes (Kobeissy et al. 2006). Prdpn inhibits apoptosis by halting the loss of mitochondrial membrane potential and reducing oxidative stress (Rodríguez et al. 2022). Sig-1R agonists lowered cell death by inhibiting caspase-3 activation (Motawe et al. 2020). The processes by which Sig-1R agonists inhibit apoptosis appear to involve the regulation of many molecular and metabolic targets, including caspase-3 cleavage.

Sig-1R may potentially regulate the production of pro- and anti-apoptotic indicators targeting mitochondria. Sig-1R activation promotes Bcl-2 expression, probably via ERK and/or nuclear factor kappa B (NF-kB) pathways (Ha et al. 2014).

GDNF and BDNF, vital neurotrophins, are instrumental in brain development, structural remodeling, neural plasticity, synaptic restructuring, and improved locomotor function following spinal cord injury via regeneration and protection of neurons (El Ouaamari et al. 2023). In our study, we found that GDNF protein and BDNF gene expression levels were considerably reduced in the spinal cord tissue following ischemic injury. This corresponded to the results of Pourkhodadad et al. and (Han et al. 2011; Pourkhodadad et al. 2021). BDNF was a principal source of neurotrophic agents and was essential for neuron survival, neuronal regeneration, and remyelination of damaged axons in spinal cord injury (Li et al. 2022).

GDNF, a member of the transforming growth factor-β family, is essential for the survival of motor and dopamine neurons. After damage, GDNF inhibits the degradation of brain cells and enhances axonal renewal and remyelination (Stanga et al. 2021; Khodir et al. 2024). BDNF and GDNF promote axonal regeneration and MPB expression in spinal cord injury (Fletcher et al. 2018).

The current study found that administering Prdpn had a neuroprotective impact and improved functional recovery in ischemia-reperfusion models of spinal cord injury by increasing GDNF and BDNF spinal expression levels. Our result aligned with prior studies which reported that Prdpn treatment upregulated the gene expression of BDNF and promoted neuronal plasticity and survival (Pourkhodadad et al. 2021). Furthermore, Prdpn can increase GDNF and BDNF expression. Thus, by acting through the Sig-1R, Prdpn facilitates functional neurorestoration in the injured nigrostriatal system (Francardo et al. 2019).

Sig-1R activation, either directly or indirectly through protein kinase C activation (Moriguchi et al. 2011), changes the phosphorylation of ERK 1/2, causing a rise in neurotrophic growth factors (Francardo et al. 2014).

Our research clarified a downregulation of Sig-1R gene expression levels in the spinal cord tissue after ischemic reperfusion injury. Zhai et al. reported that Sig-1R expression was significantly lower in a rat model of cerebral ischemia-reperfusion injury after 24 h of reperfusion (Zhai et al. 2019). Also, in a mouse model of L4-L5 rhizotomy, Sig-1R protein concentrations remained significantly lower in lumbar spinal cord sections at 7 and 14 days after injury (Gaja-Capdevila et al. 2021). The findings of our study disclosed that Prdpn treatment upregulated the Sig-1R gene expression levels in spinal cord tissues following spinal cord IRI. Animal studies exhibited that Prdpn effectively increased and restored the reduced Sig-1R levels in the HD model. Through activation of the Sig-1R, Prdpn restored mitochondrial defects caused by oxidative harm in both in vitro and ex vivo HD models (Naia et al. 2021).

The histopathological evaluation proved our results; we noticed a decline in the neuronal count within the rat model’s spinal cord tissue, along with a notable increase in the extent of demyelination and a higher incidence of apoptosis (pyknotic neurons). This was corroborated by additional research. Bahney and Bartheld affirmed that the spinal cord injury resulted in a decline in the spinal cord neuronal count in both mice and humans (Bahney and von Bartheld 2018). Additionally, studies have highlighted that spinal cord damage caused a rise in apoptosis (cell death) (Chow et al. 2023) and the extent of demyelination (loss of myelin sheath) (Shen et al. 2022). The mitochondrial pathway of apoptosis was essential, and Bax, Bcl-2, and cleaved caspase-3 were key regulators in this process (Li et al. 2015). It was found that Prdpn could mitigate neuronal cell death and apoptosis following spinal cord injury, as well as alleviating demyelination. Animal studies explained that Prdpn effectively decreased neuronal cell death and enhanced axonal transport in spinal cord explants obtained from mice with amyotrophic lateral sclerosis (Ionescu et al. 2019). Prdpn was able to regulate the Sig-1R pathway, hence offering neuroprotection to nerve cells by boosting the secretion of BDNF and GDNF (Ionescu et al. 2019).

## Conclusion

Current research is the first to show that Prdpn, a Sig-1R agonist, produces functionally relevant neuroprotective and neurorestorative influences in this spinal cord IRI rat model. The neuroprotective, anti-inflammatory, antioxidant, and anti-apoptotic properties of Prdpn on spinal cord IRI were established in this work. Administering Prdpn to rats suffering from spinal cord IRI improves the consequent neurological dysfunction by boosting the actions of endogenous antioxidant enzymes and reducing inflammatory cytokine generation, free radical formation, lipid peroxidation and apoptosis. Additionally, it increases BDNF, GDNF, and Sig-1R gene expression levels. Functional, ultrastructural, and histopathologic analyses further corroborate these findings. We believe that Prdpn could be a useful disease-modifying medication for spinal cord IRI patients, and our findings support this.

## Data Availability

The data that support the findings of this study are available from the corresponding author upon reasonable request.
